# Heart team consultations for patients with severe coronary artery disease or valvular heart disease in the era of the COVID-19 pandemic: a single-center experience

**DOI:** 10.3389/fcvm.2023.1203535

**Published:** 2023-07-19

**Authors:** Szymon Jonik, Maria Boszko, Elena Sztemberg, Dominik Łepecki, Bartłomiej Grodziński, Marcin Mikusek-Pham Van, Michał Marchel, Janusz Kochman, Mariusz Kuśmierczyk, Grzegorz Opolski, Marcin Grabowski, Tomasz Mazurek

**Affiliations:** ^1^ 1st Department of Cardiology, Medical University of Warsaw, Warsaw, Poland; ^2^ Department of Cardiac Surgery, Medical University of Warsaw, Warsaw, Poland

**Keywords:** Heart Team, COVID-19 pandemic, multivessel coronary artery disease, aortic stenosis, mitral regurgitation

## Abstract

**Introduction:**

The Heart Team (HT) as a group of experienced specialists is responsible for optimal decision-making for high-risk cardiac patients. The aim of this study was to investigate the impact of the COVID-19 pandemic on HT functioning.

**Methods:**

In this retrospective, single-center study, we evaluated the cooperation of HT in terms of the frequency of meetings, the number of consulted patients, and the trends in choosing the optimal treatment strategies for complex individuals with severe coronary artery disease (CAD) or valvular heart disease (VHD) before and during the COVID-19 pandemic in Poland.

**Results:**

From 2016 to May 2022, 301 HT meetings were held, and a total of 4,183 patients with severe CAD (2,060 patients) or severe VHD (2,123 patients) were presented. A significant decrease in the number of HT meetings and consulted patients (2019: 49 and 823 vs. 2020: 44 and 542 and 2021: 45 and 611, respectively, *P* < 0.001) as well as changes in treatment strategies—increase of conservative, reduction of invasive (2019: 16.7 and 51.9 patients/month vs. 2020: 20.4 and 24.8 patients/month and 2021:19.3 and 31.6 patients/month, respectively, *P* < 0.001)—were demonstrated with the spread of the COVID-19 pandemic. As the pandemic slowly receded, the observed changes began to return to the pre-pandemic trends.

**Conclusions:**

The COVID-19 pandemic resulted in a decrease in the number of HT meetings and consulted patients and significant reduction of invasive procedures in favor of conservative management. Further studies should be aimed to evaluate the long-term implications of this phenomenon.

## 1. Introduction

**Figure 1:**
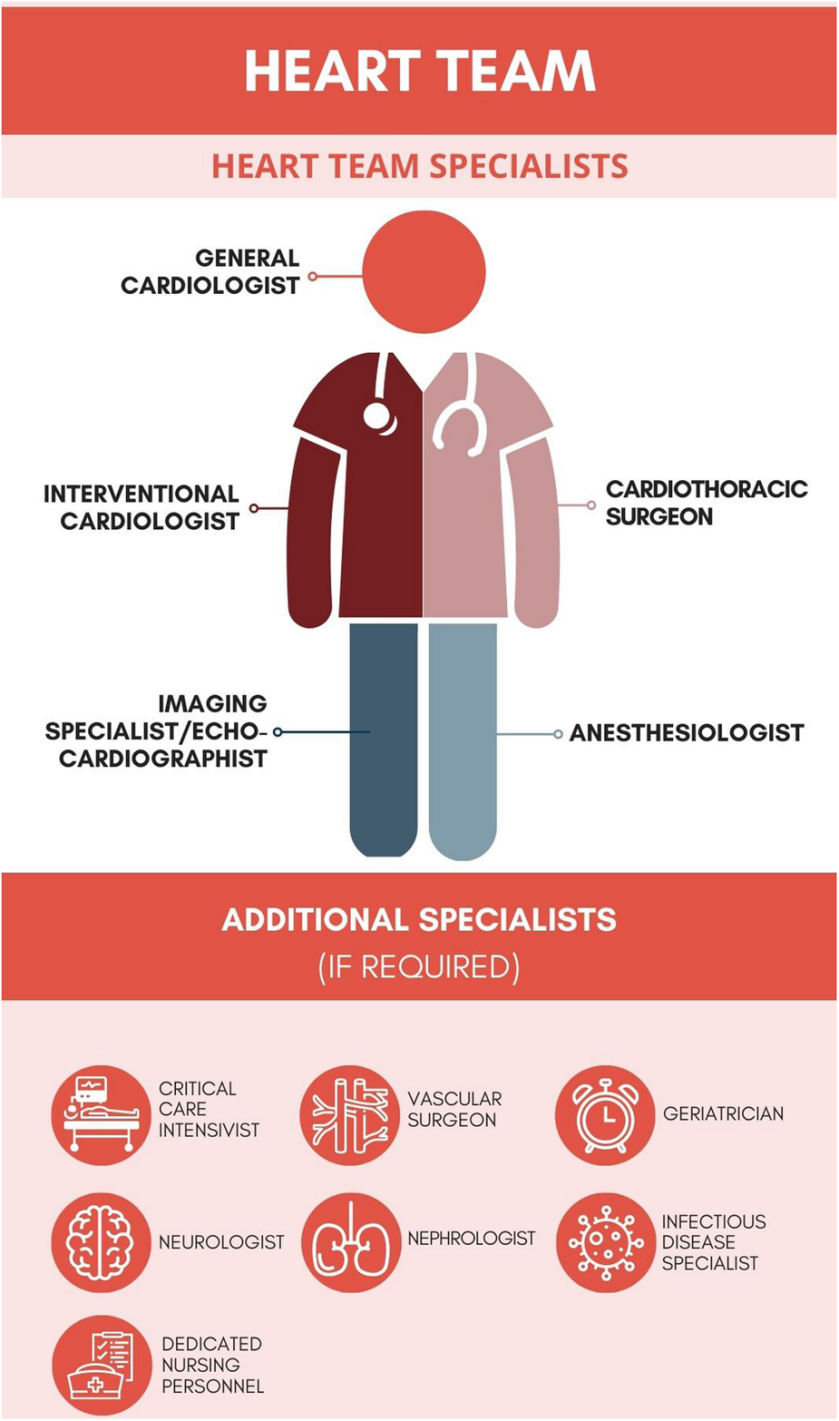
The general concept of the Heart Team.

The Heart Team (HT)—following its definition form the guidelines of the European Society of Cardiology (ESC)—is a team of experienced specialists consisting of a clinical cardiologist, a cardiothoracic surgeon, an interventional cardiologist, an imaging specialist, an anesthesiologist, and other specialists, according to current needs—Figure 1. The main objective of the HT is consensual and collective decision-making concerning high-risk cardiac patients (1). The HT tasks should be carried out during frequent meetings in accordance with clearly defined clinical supervision procedures. For many years from its concept, the idea of HT was constantly developed, but the consensus on how the HT is supposed to work, what are the goals and how HT interactions translate into real clinical practice, was unsatisfactorily described. The latest guidelines of the ESC in addition to the American Heart Association (AHA) dispel previous doubts. The documents of both organizations contain records emphasizing the essence and legitimacy of HT cooperation, placing it in class I of recommendations (1–5).

Since its outbreak in November 2019, the ongoing spread of the COVID-19 pandemic has remained a global concern. For the past 4 years, national healthcare systems worldwide have been forced to implement a multitude of changes to combat the SARS-CoV-2 infection. According to the World Health Organization (WHO), over 630 million cases and nearly 6.6 million related deaths have been reported so far. It estimated that Poland accounts for 6.35 million of diagnosed cases, as well as 118 thousand of deaths as a result of the COVID-19 (6).

Even though numerous preventive interventions regarding personal hygiene, testing, and isolating the ill were instantly undertaken, the infection spread rapidly. Initially, advocating for the minimization of contact with healthcare centers and medical professionals was aimed at limiting the further rise in the numbers of new cases and needs for hospitalizations. However, such approach ultimately led to a reluctance in seeking medical help and thus led to the development of easily preventable serious health complications among patients. Considering the accumulation of untreated cases during the pandemic, we can also expect an exponential increase in the number of patients with severe coronary artery disease (CAD) or valvular heart disease (VHD). The pandemic had also a significant impact on the general reduction of diagnostic and therapeutic interventions and contributed to several significant modifications of medical systems worldwide.

The aim of this study was to investigate the influence of the COVID-19 pandemic on the decision-making process of the multidisciplinary HT in a tertiary cardiovascular care center, with regard to frequency of meetings, number of consulted patients, and trends in choosing the optimal treatment strategies for complex individuals with CAD or VHD—aortic stenosis (AS) and mitral regurgitation (MR)—before and during the COVID-19 pandemic in Poland.

## 2. Materials and methods

This was a retrospective, cohort study conducted in the 1st Department and Clinic of Cardiology, Medical University of Warsaw, Poland, a large tertiary cardiovascular care center. The authors collected data of patients consulted for severe CAD, severe AS, and severe MR during HT meetings between 2016 and May of 2022 using electronic health records (EHR), which were then cross-checked. On 16 May 2022, the state of the COVID-19 epidemic was lifted in Poland, so we considered it appropriate to finish our analysis regarding the impact of the COVID-19 pandemic on the functioning of the HT in May 2022. The inclusion criteria for patients presented during HT meetings were as follows: aged ≥18 years and complete clinical, echocardiographic, and angiographic characteristics. Furthermore, the angiographic and echocardiographic inclusion criteria for severe CAD, severe AS, and severe MR were reported previously (7–9). The optimal medical therapy (OMT) was defined as using of drugs with proven impact on increased survival or providing optimal reduction of the signs and symptoms associated with CAD or VHD. Further assessment (FA) was defined as the implementation of additional non-invasive cardiac diagnostic tests and imaging studies or evaluation of clinical symptoms and echocardiographic parameters within 3–6 months from the initial diagnosis. The criteria for excluding patients from the final analysis included the following: pregnancy/lactation, disseminated neoplastic process, life expectancy <1 year, or lack of informed, written consent. The definitions for baseline characteristics and echocardiographic and angiographic parameters were reported previously (7–9). All patients were evaluated in weekly meetings by an HT composed of at least an interventional cardiologist, cardiac surgeon, clinical cardiologist, and non-invasive imaging specialist.

After the HT review and discussion, the patients were qualified to one of the three main treatment strategies: surgical, percutaneous, or conservative—for CAD—coronary artery bypass grafting (CABG), percutaneous coronary intervention (PCI), optimal medical treatment (OMT), or FA; for AS—surgical aortic valve replacement (SAVR), transcatheter aortic valve replacement (TAVR), or OMT/FA; and for MR—mitral valve replacement/mitral valve repair (MVR/MVP), transcatheter edge-to-edge repair (TEER), or OMT/FA. The data regarding the number of HT meetings, number of consulted patients, and trends in choosing the optimal treatment strategies were collected retrospectively. The institutional review board (IRB) was informed. Considering the retrospective, observational nature of this study, no further approval was necessary.

### 2.1. Statistical analysis

The PQStat software (version 1.6.6, PQStat, Poznań, Poland) was used for the statistical analysis. The normality of distribution for continuous variables was confirmed with the Shapiro–Wilk test. The categorical data were expressed as counts and percentages, while continuous data were presented as mean ± SD. The comparison between groups of patients qualified for individual treatment strategies was performed using chi-square test, and the statistical analysis was executed using one-way analysis of variance (ANOVA). All *P*-values (*P*) were given to at least two-sided, and *P*-value lower than 0.05 were considered statistically significant.

## 3. Results

### 3.1. Patient characteristics

Between 2016 and May of 2022, a total of 4,183 patients with severe CAD (*n* = 2,060), severe AS (*n* = 1,528), or severe MR (*n* = 595) were presented during 301 HT meetings with a mean of 13.9 patients presented per meeting. The baseline clinical characteristics of each cohort along with treatment strategies (surgical, percutaneous, or conservative) are presented in Tables 1–3.

**Table 1:** Patients with severe coronary artery disease (CAD) consulted by the Heart Team (HT) in 2016–2022, May—baseline characteristics.

Baseline characteristics	Overall (2,060)	CABG (533)	PC (1,019)	OMT (371)	Further assessment (137)	*P*-value
Age, years [mean (SD)]	69.2 (9.8)	66.8 (9.2)	69.1 (10.1)	72.3 (9.9)	69.9 (9.6)	<0.001
Gender, male [*n* (%)]	1,561 (75.8)	435 (81.6)	747 (73.3)	265 (71.4)	114 (83.2)	<0.001
BMI, kg/m^2^ [mean (SD)]	27.9 (3.5)	27.6 (3.4)	28.3 (4.0)	26.9 (3.1)	28.5 (3.8)	<0.001
Heart failure [*n* (%)]	1,556 (75.5)	353 (66.2)	750 (73.6)	342 (92.2)	111 (81.0)	<0.001
NYHA class III–IV [*n* (%)]	696 (33.8)	142 (26.6)	323 (31.7)	190 (51.2)	41 (29.9)	<0.001
LVEF, % [mean (SD)]	37.5 (10.5)	39.0 (10.6)	36.9 (10.7)	35.1 (10.3)	41.4 (9.5)	<0.001
LVEDD [cm (SD)]	5.7 (1.0)	5.4 (0.9)	5.7 (1.1)	5.9 (0.9)	5.8 (1.0)	<0.001
CCS class III–IV [*n* (%)]	850 (41.3)	249 (46.7)	403 (39.6)	132 (35.6)	66 (48.2)	<0.001
Diabetes [*n* (%)]	637 (30.9)	146 (27.4)	329 (32.3)	112 (30.2)	50 (36.5)	0.11
Hypertension [*n* (%)]	1,697 (82.4)	437 (82.0)	851 (83.5)	297 (80.1)	112 (81.8)	0.50
Previous stroke/TIA [*n* (%)]	181 (8.8)	38 (7.1)	90 (8.8)	41 (11.1)	12 (8.8)	0.24
Atrial fibrillation [*n* (%)]	569 (27.6)	101 (19.0)	283 (27.8)	140 (37.7)	45 (32.9)	<0.001
Previous MI [*n* (%)]	996 (48.4)	283 (53.1)	479 (47.0)	159 (42.9)	75 (54.7)	<0.001
Previous PCI [*n* (%)]	623 (30.2)	141 (26.5)	326 (32.0)	109 (29.4)	47 (34.3)	0.10
Previous CABG [*n* (%)]	187 (9.1)	34 (6.4)	106 (10.4)	34 (9.2)	13 (9.5)	0.08
PAD [*n* (%)]	127 (6.2)	21 (3.9)	73 (7.2)	22 (5.9)	11 (8.0)	0.07
CKD [*n* (%)]	751 (36.5)	98 (18.4)	306 (30.0)	285 (76.8)	62 (45.3)	<0.001
Anemia [*n* (%)]	811 (39.4)	133 (25.0)	364 (35.7)	232 (62.5)	82 (59.9)	<0.001
Dyslipidemia [*n* (%)]	1,656 (80.4)	425 (79.7)	835 (81.9)	283 (76.3)	113 (82.5)	0.11
COPD [*n* (%)]	209 (10.2)	43 (8.1)	105 (10.3)	44 (11.9)	17 (12.4)	0.21
Severe PH [*n* (%)]	202 (9.8)	26 (4.9)	110 (10.8)	52 (14.0)	14 (10.2)	<0.001
Cancer [*n* (%)]	348 (16.9)	33 (6.2)	156 (15.3)	131 (35.3)	28 (20.4)	<0.001
Smoking [*n* (%)]	379 (18.4)	98 (18.4)	199 (19.5)	55 (14.8)	27 (19.7)	0.24
Frailty [*n* (%)]	423 (20.5)	13 (2.4)	134 (13.2)	241 (65.0)	35 (25.6)	<0.001
No. of lesions [mean (SD)]	4.2 (1.5)	4.2 (1.4)	4.3 (1.6)	4.1 (1.5)	4.2 (1.6)	0.43
SYNTAX score [mean (SD)]	30.4 (6.4)	31.4 (6.0)	29.8 (6.6)	30.3 (6.4)	30.7 (6.4)	0.17
EuroSCORE II, % [mean (SD)]	5.6 (3.1)	3.9 (1.2)	6.1 (3.9)	6.6 (4.0)	5.9 (2.5)	<0.001
STS score, % [mean (SD)]	3.7 (1.7)	2.6 (0.9)	4.0 (2.1)	4.2 (1.9)	3.6 (1.7)	<0.001

BMI, body mass index; CABG, coronary artery bypass grafting; CCS, Canadian Cardiovascular Society; CKD, chronic kidney disease; COPD, chronic obstructive pulmonary disease; EF, ejection fraction; EuroSCORE II, European System for Cardiac Operative Risk Evaluation II; LVEDD, left ventricular end-diastolic diameter; MI, myocardial infarction; NYHA, New York Heart Association; OMT, optimal medical treatment; PAD, peripheral arterial disease; PCI, percutaneous coronary intervention; PH, pulmonary hypertension; STS, Society of Thoracic Surgeons; SYNTAX score, synergy between percutaneous coronary intervention with Taxus and cardiac surgery score; TIA, transient ischemic attack.

**Table 2:** Patients with severe aortic stenosis (AS) consulted by the Heart Team (HT) from 2016 to 2022, May—baseline characteristics.

Baseline characteristics	Overall (1,528)	TAVR (907)	SAVR (366)	OMT (161)	Further assessment (94)	*P*-value
Age, years [mean (SD)]	78.0 (7.2)	80.3 (7.4)	70.9 (6.0)	81.4 (7.8)	77.2 (8.5)	<0.001
Gender, male [*n* (%)]	726 (47.5)	425 (46.9)	192 (52.5)	67 (41.6)	42 (44.7)	0.10
BMI, kg/m^2^ [mean (SD)]	28.0 (4.9)	28.5 (5.1)	27.7 (4.0)	25.9 (5.5)	27.8 (5.2)	<0.001
Heart failure [*n* (%)]	1,186 (77.6)	781 (86.1)	216 (59.0)	148 (91.9)	41 (43.6)	<0.001
NYHA class III–IV [*n* (%)]	745 (48.8)	527 (58.1)	89 (24.3)	103 (64.0)	26 (27.7)	<0.001
LVEF, % [mean (SD)]	50 (12.7)	51 (13.2)	52 (12.2)	41 (12.6)	48 (9.8)	<0.001
AVA, cm^2^ [mean (SD)]	0.83 (0.20)	0.83 (0.19)	0.79 (0.21)	0.81 (0.22)	1.04 (0.26)	<0.001
AVA I, cm^2^/m^2^ [mean (SD)]	0.48 (0.20)	0.47 (0.19)	0.45 (0.20)	0.53 (0.20)	0.66 (0.24)	<0.001
CAD [*n* (%)]	789 (51.6)	478 (52.7)	170 (46.4)	92 (57.1)	49 (52.1)	0.10
Diabetes [*n* (%)]	540 (35.3)	342 (37.7)	99 (27.1)	68 (42.2)	31 (33.0)	<0.001
Hypertension [*n* (%)]	1,317 (86.2)	787 (86.8)	311 (85.0)	140 (87.0)	79 (84.0)	0.76
Previous stroke/TIA [*n* (%)]	168 (11.0)	86 (9.5)	18 (4.9)	46 (28.6)	18 (19.2)	<0.001
Atrial fibrillation [*n* (%)]	459 (30.0)	292 (32.2)	91 (24.9)	49 (30.4)	27 (28.7)	0.08
Previous MI [*n* (%)]	368 (24.1)	221 (24.4)	75 (20.5)	50 (31.1)	22 (23.4)	0.07
Previous PCI [*n* (%)]	614 (40.2)	382 (42.1)	127 (34.7)	69 (42.9)	36 (38.3)	0.09
Previous CABG [*n* (%)]	174 (11.4)	99 (10.9)	35 (9.6)	28 (17.4)	12 (12.8)	0.06
Previous non-aortic VS [*n* (%)]	83 (5.4)	45 (5.0)	16 (4.4)	13 (8.1)	9 (9.6)	0.09
History of pacemaker [*n* (%)]	281 (18.4)	181 (20.0)	9 (2.5)	73 (45.3)	18 (19.2)	<0.001
PAD [*n* (%)]	276 (18.1)	149 (16.4)	37 (10.1)	78 (48.5)	12 (12.8)	<0.001
CKD [*n* (%)]	923 (60.4)	638 (70.3)	127 (34.7)	126 (78.3)	32 (34.0)	<0.001
Anemia [*n* (%)]	852 (55.8)	597 (65.8)	84 (23.0)	134 (83.2)	37 (39.4)	<0.001
Dyslipidemia [*n* (%)]	1,173 (76.8)	707 (78.0)	275 (75.1)	120 (74.5)	71 (75.5)	0.62
COPD [*n* (%)]	206 (13.5)	120 (13.2)	26 (7.1)	49 (30.4)	11 (11.7)	<0.001
Severe PH [*n* (%)]	125 (8.2)	60 (6.6)	11 (3.0)	45 (28.0)	9 (9.6)	<0.001
Cancer [*n* (%)]	196 (12.8)	112 (12.4)	29 (7.9)	43 (26.7)	12 (12.8)	<0.001
Smoking [*n* (%)]	1,021 (66.8)	616 (67.9)	232 (63.4)	110 (68.3)	63 (67.0)	0.46
Frailty [*n* (%)]	546 (35.7)	386 (42.6)	21 (5.7)	110 (68.3)	19 (20.2)	<0.001
EuroSCORE II, % [mean (SD)]	8.5 (9.1)	9.4 (11.0)	4.9 (4.0)	11.7 (10.6)	8.1 (7.3)	<0.001
STS score, % [mean (SD)]	5.6 (4.9)	6.1 (5.0)	3.5 (2.1)	7.2 (5.6)	5.6 (4.7)	<0.001

AVA, aortic valve area; AVA I, indexed aortic valve area; BMI, body mass index; CABG, coronary artery bypass grafting; CAD, coronary artery disease, CCS, Canadian Cardiovascular Society; CKD, chronic kidney disease; COPD, chronic obstructive pulmonary disease; EF, ejection fraction; EuroSCORE II, European System for Cardiac Operative Risk Evaluation II; MI, myocardial infarction; NYHA, New York Heart Association; OMT, optimal medical treatment; PAD, peripheral arterial disease; PCI, percutaneous coronary intervention; PH, pulmonary hypertension; SAVR, surgical aortic valve replacement; STS, Society of Thoracic Surgeons; SYNTAX score, Synergy between percutaneous coronary intervention with Taxus and cardiac surgery score; TAVR, transcatheter aortic valve replacement; TIA, transient ischemic attack; VS, valvular surgery.

**Table 3:** Patients with severe mitral regurgitation (MR) consulted by heart team (HT) from 2016 to 2022, May—baseline characteristics.

Baseline characteristics	Overall (595)	TEER (153)	MVR/MVP (195)	OMT (153)	Further assessment (94)	*P*-value
Age, years [mean (SD)]	72.2 (9.3)	73.8 (9.1)	68.4 (8.9)	74.6 (10.0)	73.5 (9.4)	<0.001
Gender, male [*n* (%)]	347 (58.3)	83 (54.3)	120 (61.5)	90 (58.8)	54 (57.5)	0.59
BMI, kg/m^2^ [mean (SD)]	26.5 (5.6)	26.2 (5.5)	26.9 (5.7)	25.9 (5.5)	27.2 (6.0)	0.06
Heart failure [*n* (%)]	556 (93.4)	153 (100.0)	177 (90.8)	150 (98.0)	76 (80.9)	<0.001
NYHA class III–IV [*n* (%)]	349 (58.7)	112 (73.2)	102 (52.3)	95 (62.1)	40 (42.6)	<0.001
LVEF, % [mean (SD)]	37.2 (9.4)	34.1 (8.5)	39.9 (10.1)	34.7 (8.9)	40.8 (10.3)	<0.001
ERO, cm^2^ [mean (SD)]	0.44 (0.20)	0.42 (0.19)	0.44 (0.20)	0.49 (0.22)	0.39 (0.18)	<0.001
Regurgitant volume, ml/beat [mean (SD)]	47.3 (18.4)	47.6 (19.1)	48.3 (18.4)	49.5 (18.6)	41.4 (16.9)	<0.001
CAD [*n* (%)]	339 (57.0)	99 (64.7)	91 (46.7)	101 (66.0)	48 (51.1)	<0.001
Diabetes [*n* (%)]	239 (40.2)	67 (43.8)	65 (33.3)	65 (42.5)	42 (44.7)	0.12
Hypertension [*n* (%)]	540 (90.8)	142 (92.8)	177 (90.8)	136 (88.9)	85 (90.4)	0.70
Previous stroke/TIA [*n* (%)]	121 (20.3)	42 (27.5)	21 (10.8)	46 (30.1)	12 (12.8)	<0.001
Atrial fibrillation [*n* (%)]	273 (45.9)	74 (48.4)	77 (39.5)	79 (51.6)	43 (45.7)	0.13
Previous MI [*n* (%)]	239 (40.2)	78 (51.0)	51 (26.2)	74 (48.4)	36 (38.3)	<0.001
Previous PCI [*n* (%)]	296 (49.7)	90 (58.8)	68 (34.9)	94 (61.4)	44 (46.8)	<0.001
Previous CABG [*n* (%)]	80 (13.4)	23 (15.0)	16 (8.2)	26 (17.0)	15 (16.0)	0.07
PKD [*n* (%)]	132 (22.2)	37 (24.2)	31 (15.9)	40 (26.1)	24 (25.5)	0.08
CKD [*n* (%)]	450 (75.6)	142 (92.8)	106 (54.4)	147 (96.1)	55 (58.5)	<0.001
Anemia [*n* (%)]	422 (70.9)	139 (90.9)	84 (43.1)	142 (92.8)	57 (60.6)	<0.001
Dyslipidemia [*n* (%)]	517 (86.9)	134 (87.6)	167 (85.6)	136 (88.9)	80 (85.1)	0.77
COPD [*n* (%)]	111 (18.7)	36 (23.5)	16 (8.2)	39 (25.5)	20 (21.3)	<0.001
Severe PH [*n* (%)]	97 (16.3)	38 (24.8)	11 (5.6)	43 (28.1)	21 (22.3)	<0.001
Cancer [*n* (%)]	116 (19.5)	35 (22.9)	17 (8.7)	45 (29.4)	19 (20.2)	<0.001
Smoking [*n* (%)]	421 (70.8)	112 (73.2)	137 (70.3)	109 (71.2)	63 (67.0)	0.77
Frailty [*n* (%)]	164 (27.6)	53 (34.6)	6 (3.1)	74 (48.4)	31 (33.0)	<0.001
EuroSCORE II, % [mean (SD)]	8.3 (7.4)	9.8 (8.2)	5.4 (3.7)	10.9 (8.7)	7.9 (5.1)	<0.001
STS score, % [mean (SD)]	5.5 (4.1)	6.5 (5.2)	3.6 (2.4)	7.2 (5.7)	5.2 (3.4)	<0.001

BMI, body mass index; CABG, coronary artery bypass grafting; CAD, coronary artery disease, CCS, Canadian Cardiovascular Society; CKD, chronic kidney disease; COPD, chronic obstructive pulmonary disease; EF, ejection fraction; EuroSCORE II, European System for Cardiac Operative Risk Evaluation II; ERO, effective regurgitant orifice; LVEDD, left ventricular end-diastolic diameter; MI, myocardial infarction; MVR/MVP, mitral valve replacement/mitral valve repair; NYHA, New York Heart Association; OMT, optimal medical treatment; PAD, peripheral arterial disease; PCI, percutaneous coronary intervention; PH, pulmonary hypertension; STS, Society of Thoracic Surgeons; SYNTAX score, synergy between percutaneous coronary intervention with Taxus and cardiac surgery score; TEER, transcatheter edge-to-edge repair; TIA, transient ischemic attack.

### 3.2. CAD cohort

A total of 2,060 individuals from the CAD cohort [1,561 (75.8%) male, age [years, mean (SD)] = 69.2 (9.8), EuroSCORE II (European System for Cardiac Operative Risk Evaluation II), %; mean (SD) = 5.6 (3.1), STS (Society of Thoracic Surgeons) score, %; mean (SD) = 3.7 (1.7), and given comorbidities] were assigned by HT to treatment modalities as follows: CABG—533, PCI—1,019, OMT—371, and FA—137. Briefly, regarding statistically significant differences between treatment strategies, the patients that qualified for OMT were significantly older and more frail, while the percentage of male and body mass index (BMI) were the lowest in this group. The OMT participants presented more often with heart failure (HF), reduced left ventricle ejection fraction (LVEF), increased left ventricle end-diastolic diameter (LVEDD), and symptoms in the New York Heart Association (NYHA) class III–IV were often burdened with atrial fibrillation (AF), chronic kidney disease (CKD), anemia, severe pulmonary hypertension (PH), and cancer and with the highest perioperative risk of intervention assessed both by the EuroSCORE II and STS than those that qualified for CABG, PCI, or FA (*P* < 0.001). The individuals that qualified for CABG or FA had the most severe symptoms of CAD (CCS class III–IV), and also the history of previous myocardial infarction (MI) was the most frequent in these groups, *P* < 0.001—Table 1.

### 3.3. AS cohort

A total of 1,528 patients from the AS cohort [726 (47.5%) male, age [years, mean (SD)] = 78.0 (7.2), EuroSCORE II, %; mean (SD) = 8.5 (9.1), STS score, %; mean (SD) = 5.6 (4.9) and given comorbidities] were allocated by HT to treatment strategies as follows: SAVR—366, TAVR—907, OMT—161, and FA—94. As regards statistically significant differences between TAVR, SAVR, OMT, and FA groups, the patients that qualified for OMT were the oldest, more often frail, and with the lowest BMI. They presented more often with HF, reduced LVEF and symptoms in NYHA III–IV class, were often burdened with diabetes, peripheral artery disease (PAD), CKD, anemia, chronic obstructive pulmonary disease (COPD), severe PH and cancer, with history of previous stroke/transient ischemic attack (TIA) and implanted pacemaker, and with the highest perioperative risk of intervention assessed both by the EuroSCORE II and STS than those that qualified for SAVR, TAVR, or FA (*P* < 0.001). The prevalence of CAD, history of previous MI, PCI, and CABG, were similar between groups. The aortic valve area (AVA, cm^2^) and indexed AVA (cm^2^/m^2^) were the lowest in the SAVR group (*P* < 0.001). The baseline clinical characteristics (overall and by groups) in details are presented in Table 2.

### 3.4. MR cohort

A total of 595 individuals from the MR cohort [347 (58.3%) male, age [years, mean (SD)] = 72.2 (9.3), EuroSCORE II, %; mean (SD) = 8.3 (7.4), STS score, %; mean (SD) = 5.5 (4.1) and given comorbidities] were assigned by HT to treatment modalities as follows: MVR/MVP—195, TEER—153, OMT—153, and FA—94. The patients from the surgical group were statistically the youngest and with the lowest perioperative risk of intervention assessed both by the EuroSCORE II and STS (*P* < 0.001). Those allocated for TEER or OMT were the most burdened and frail, and the rates of HF, NYHA class III–IV symptoms, CAD and coronary events (MI and PCI), previous stroke/TIA, CKD, anemia, COPD, severe PH, and history of cancer were the highest in these groups, while the LVEFs were the most reduced. The MR parameters: ERO (effective regurgitant orifice, cm^2^) and regurgitant volume (ml/beat) were the least severe in the FA group (*P* < 0.001). The details of baseline characteristics of the MR cohort were presented in Table 3.

### 3.5. Number of patients, frequency of meeting, trends in the chosen treatment strategies

**Figure 2:**
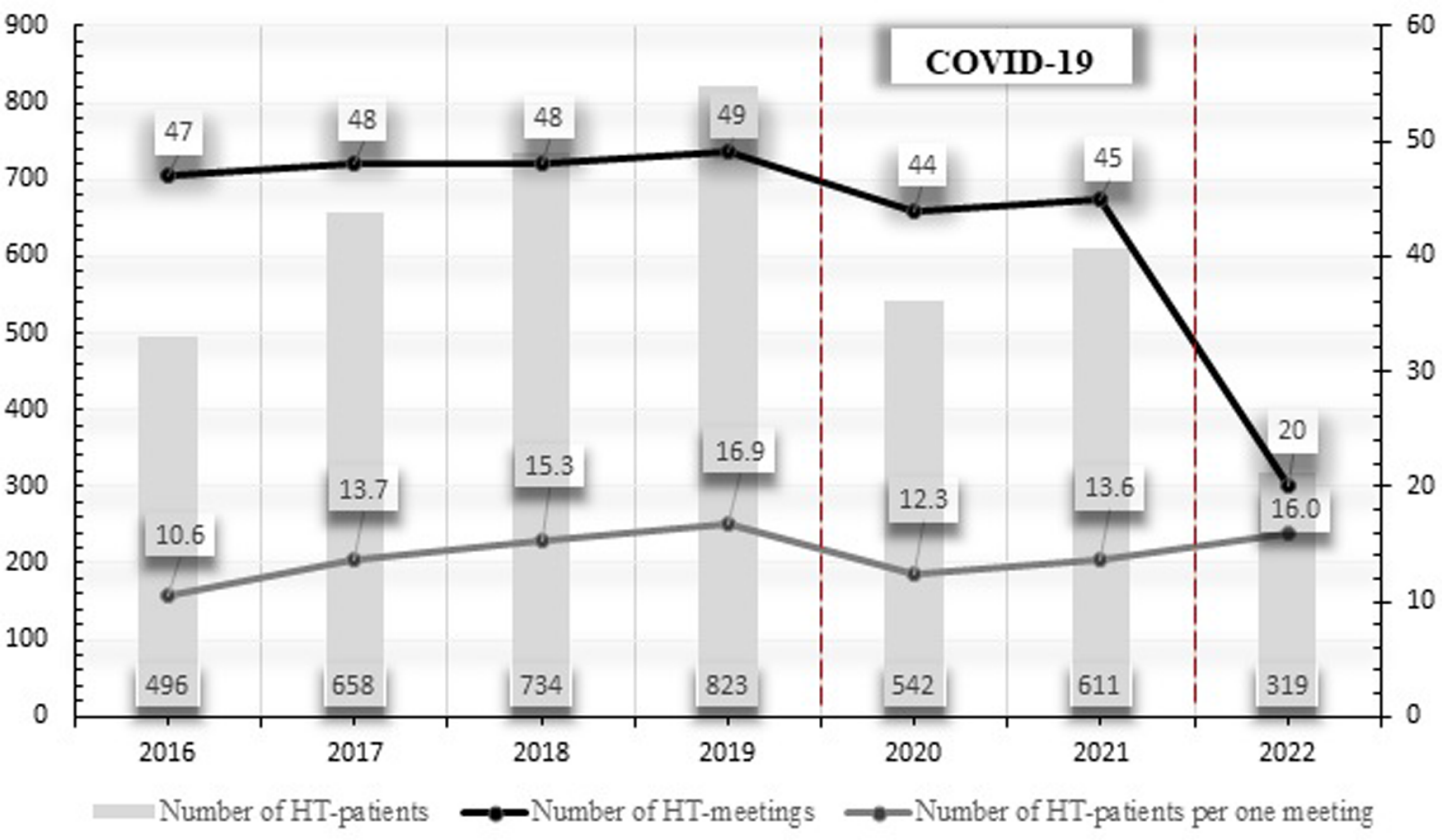
Trends in the number of HT patients, number of HT meetings, and number of HT patients per one meeting in the years 2016–2022, May.

**Figure 3:**
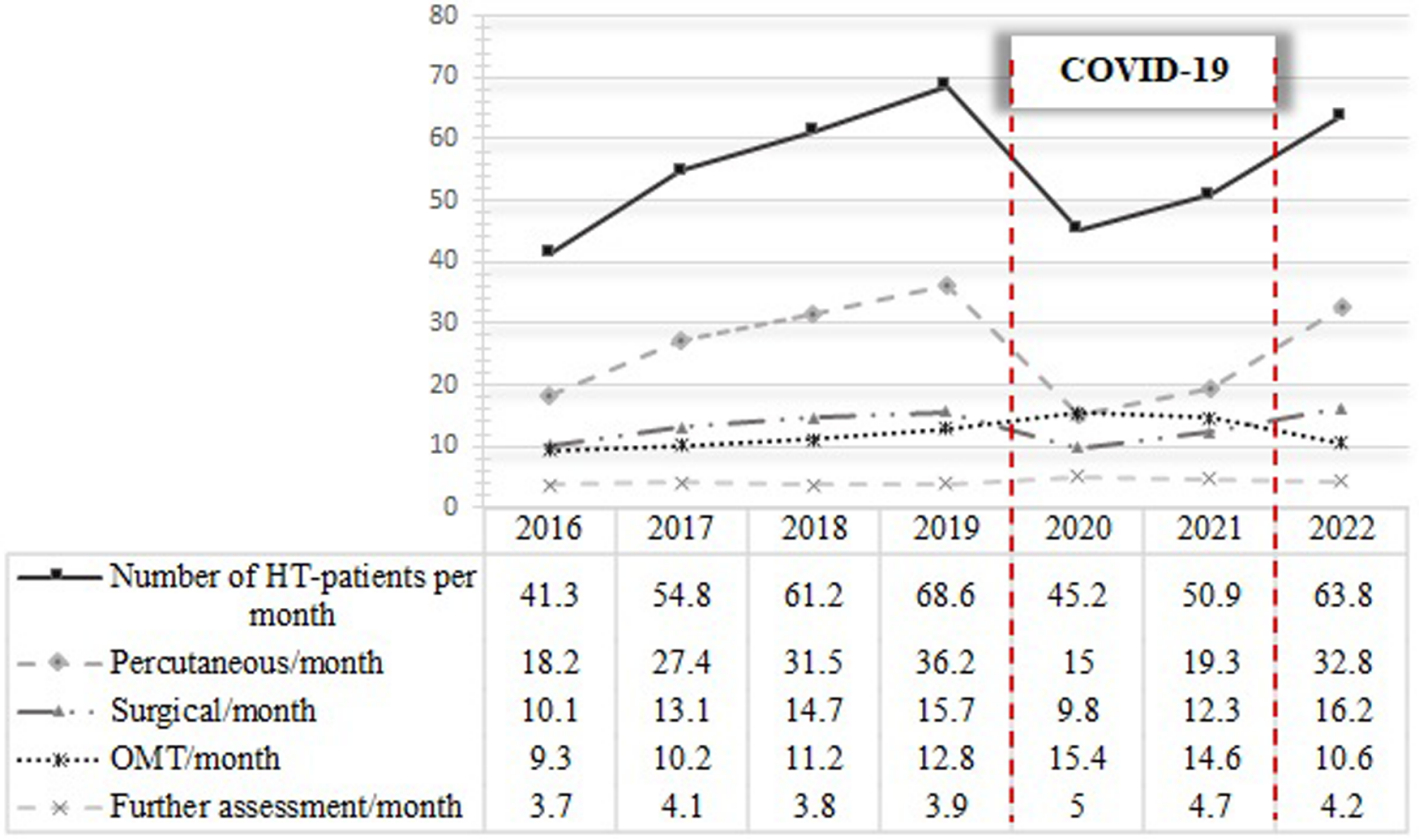
Trends in the number of monthly consulted HT patients and changes in selection of treatment strategies in years 2016–2022, May. Influence of the COVID-19 pandemic.

With the spread of the COVID-19 pandemic, a significant change in the number of HT meetings, number of consulted patients, and the chosen treatment strategies were observed. Compared to the pre-pandemic time, during which a relatively constant year-to-year rate of HT meetings was demonstrated, in 2020 and 2021, we noticed a significant decrease in the frequency of annual sessions (2019—49 vs. 2020—44 and 2021—45, respectively, *P* < 0.001) (Figure 2). Furthermore, the number of consulted patients (overall and per meeting) has also reduced (2019—823 and 16.8 vs. 2020—542 and 12.3 and 2021—611 and 13.6 patients and patients per meeting, respectively, *P* < 0.001) (Figure 2). Comparing the results of our qualifications with the pre-pandemic time, a significant change in the chosen treatment modalities was also demonstrated. During the COVID-19 pandemic, we qualified fewer patients to interventional strategies—both surgical and percutaneous (2019—51.9 vs. 2020—24.8 and 2021—31.6 patients per month, respectively, *P* < 0.001), while significantly more individuals were assigned to conservative approach—OMT or FA (2019—16.7 vs. 2020—20.4 and 2021—19.3 patients per month, respectively, *P* < 0.001) (Figure 3 and Table 4). As the pandemic slowly receded until the end of the state of the COVID-19 epidemic in Poland (the first 5 months of 2022), the described changes began to return to the pre-pandemic trends (Figures 2, 3 and Table 4).

**Table 4:** Selected treatment strategies for patients consulted by the Heart Team (HT) in years 2016–2022, May.

MVD		AS		MR	
Overall, *n*	2,060	Overall, *n*	1,528	Overall, *n*	595
CABG, *n* (*n*/per month)	533 (6.9/month)	SAVR, *n* (*n*/per month)	366 (4.8/month)	MVR/MVP, *n* (*n*/per month)	195 (2.5/month)
PCI, *n* (*n*/per month)	1,019 (13.2/month)	TAVR, *n* (*n*/per month)	907 (11.8/month)	TEER, *n* (*n*/per month)	153 (2.0/month)
OMT, *n* (*n*/per month)	371 (4.8/month)	OMT, *n* (*n*/per month)	161 (2.1/month)	OMT, *n* (*n*/per month)	153 (2.0/month)
Further assessment, *n* (*n*/per month)	137 (1.8/month)	Further assessment, *n* (*n*/per month)	94 (1.2/month)	Further assessment, *n* (*n*/per month)	94 (1.2/month)

AS, aortic stenosis; CABG, coronary artery bypass grafting; MR, mitral regurgitation; MVD, multivessel disease; MVR/MVP, mitral valve replacement/mitral valve repair; OMT, optimal medical treatment; PCI, percutaneous coronary intervention; SAVR, surgical aortic valve replacement; TAVR, transcatheter aortic valve replacement; TEER, transcatheter edge-to-edge repair.

## 4. Discussion

Evidence keeps growing that the multidisciplinary approach of the HT in the management of CAD and VHD patients results in better outcomes. This belief is notwithstanding based exclusively on the data from some observational studies and opinions of the experts. Therefore, we call for further research in this area. As mentioned before, the Heart Team consists of a group of experienced physicians, representing numerous specialties (Figure 1). The collective decision-making process ensures that all potential benefits and risks are thoroughly discussed before meeting a final decision regarding the treatment of a patient. Considering the number of involved physicians, a more holistic, yet individual, approach can be adapted. This is especially relevant for high-risk cardiac patients, in whom several variables (anatomical, clinical, and procedural characteristics) should be taken into account beforehand. The COVID-19 pandemic has raised additional challenges for the HT members. After carefully evaluating the data of the patient, the epidemiological circumstances were often the final determining factor. Nevertheless, sustaining the HT workflow in the pandemic reality provided the patients with crucial continuity of care.

Owing to the COVID-19 pandemic, several aspects of HT functioning had to be transformed in order to adapt to the new reality. In some centers, the majority of clinical and imaging data were transmitted electronically, aiming to minimize the necessity of face-to-face meetings. Furthermore, phone calls were used whenever possible to avoid direct contact between the consulting physicians. After a conclusive decision has been made, the recommendations of the HT were also transferred back electronically. In addition, considering the poor prognosis of conservatively treated patients, applying close-out patient monitoring based on telemedicine has been strongly advised for those who are considered hemodynamically stable and whose procedures could be postponed safely (10–12). Therefore, the use of telemedicine and other virtual aspects of communication in the era of the COVID-19 pandemic have proven invaluable.

One of the major findings of our study is the decrease in the number of HT meetings and lower numbers of consulted patients during the peak of the COVID-19 pandemic. It is very likely that the cause of this phenomenon is complex. There are several studies demonstrating that one of the factors associated with lower admission rates to emergency department (ED) and a decrease in follow-up (FU) visits was the fear of being infected with SARS-CoV-2 (13, 14). In addition, those patients whose CAD, AS, or MR-related symptoms worsened during the pandemic were more anxious, more likely to cancel their scheduled appointments or waited longer before reporting to the ED. Similar observations have been made with regard to patients with acute coronary syndrome (ACS) in a study conducted in Italy (15). In 2020, at the peak of the pandemic, a significant shift from the guidelines-recommended invasive strategies toward a more conservative approach was observed. Mohamed et al. also reported a noticeable decrease of all major cardiac procedures as a result of the COVID-19 pandemic in England, with CABG, along with valvular interventions listed as the most affected procedures, which is rather similar to our results (16). Summarizing the above, we conclude that consulting patients could have been more difficult and time-consuming. During the COVID-19 era, scheduled admissions have been limited. According to opinions of the experts, to ensure the continuity of optimal care as well as prevent an increased risk of infection, appropriate triage of patients was necessary; hence, those with severe symptomatic disease were given priority (10, 11). All individuals had to be tested for SARS-CoV-2 infection. Invasive procedures were postponed, if possible, until the COVID-19 was confirmed or excluded, which usually extended the diagnostic or therapeutic process by at least 12–24 h, except for life-saving indications.

As a result of postponing the decisions on whether or not to perform the intervention, the COVID-19 pandemic has affected the patients with severe conditions both directly and indirectly. While waiting, the primary illness of the patient frequently further deteriorated, as it was believed that the COVID-19 infection should be treated first, in the absence of other life-threatening diseases. Limiting the admissions and interventions, especially at the very beginning of the COVID-19 pandemic, was forced by staff shortages, especially anesthesiologists, who were delegated to work in the Intensive Care Units (ICUs). Another factor that affected the number and scope of interventions during the COVID-19 era was the obligation to wear personal protective equipment (PPE). It has contributed to the prolongation of procedure duration and was associated with special proceedings to manage the patients properly (15). Furthermore, some studies demonstrated that the use of PPE was directly associated with the effectiveness of the work of surgeons and cardiologists, negatively affected communication, visibility, and situational awareness and caused increased fatigue, but with no statistically significant impact on their technical skills (17).

After the outbreak of the COVID-19 pandemic, the need to limit the potential exposure of medical personnel (including highly specialized cardiologists and cardiac surgeons) was highlighted as a key priority in the European guidelines (18, 19). The reduction of invasive procedures may have also resulted from the desperate need to save valuable resources, including PPEs, pharmaceuticals, ICU beds, ventilators, or extracorporeal membrane oxygenation (ECMO) devices (20). Other factors may have been related to the desired minimization of the ICU burden and the expected shortening of the hospital stay. Therefore, it was recommended to favor such proceeding in patients who can be monitored (asymptomatic, hemodynamically stable) and in whom intervention could be safely delayed. It is worth mentioning that echocardiography, which for patients with VHD is the basis for making the diagnosis and monitoring the abnormal parameters, should be considered as a potentially dangerous, aerosol-generating procedure (10, 21). Nevertheless, the poor prognosis of OMT patients, priority should be given to interventional strategies, according to local and current epidemiological conditions.

Owing to the necessity of social distancing, telemedicine has become a field of extensive research during the COVID-19 pandemic (22–24). Various virtual methods of communication were explored to ensure the continuity of the medical workflow. Notwithstanding, in many cases, digital solutions are still not widely recommended, and the implementation of such novel approach in everyday clinical practice remains a challenge (25). Utilizing telemedicine by the HT to maintain appropriate care has been strongly suggested in the previous guidelines (10). Broader implementation of this novel approach for conducting HT meetings should be further discussed and investigated in the future. However, virtual consultations should be initiated cautiously with their possible limitations taken into account (24–27). The main ones include the following: the likelihood of lower attendance during HT meetings and technical difficulties—insufficient quality of transmitted images or poor online connection, which may have a significant impact on selecting optimal management. Difficulty of expressing oneself due to lack of prior experience with telemedicine should be also considered. Another case is that the digital perceptions and experiences may differ from those during in-person meetings, which could also affect the discussion and decision-making process. Regardless of the direction in which further functioning and the concept of HT will develop, it should be noted that the unexpected COVID-19 pandemic revealed numerous problems of the medical care systems and forced the implementation of some innovative medical solutions that had been planned for years.

## Data Availability

The raw data supporting the conclusions of this article will be made available by the authors, without undue reservation.
